# Therapeutic potential of mesenchymal stem cells for fungal infections: mechanisms, applications, and challenges

**DOI:** 10.3389/fmicb.2025.1554917

**Published:** 2025-01-30

**Authors:** Yangjie Gao, Zhe Ji, Jingyu Zhao, Julin Gu

**Affiliations:** ^1^Department of Dermatology, Third Affiliated Hospital of Naval Medical University, Shanghai, China; ^2^Department of Pharmacology, Shanghai Tenth People’s Hospital, School of Medicine, Tongji University, Shanghai, China

**Keywords:** mesenchymal stem cells, fungal infections, therapeutic potential, mechanisms, applications

## Abstract

As a particularly serious condition in immunocompromised patients, fungal infections (FIs) have increasingly become a public health problem worldwide. Mesenchymal stem cells (MSCs), characterized by multilineage differentiation potential and immunomodulatory properties, are considered an emerging strategy for the treatment of FIs. In this study, the therapeutic potential of MSCs for FIs was reviewed, including their roles played by secreting antimicrobial peptides, regulating immune responses, and promoting tissue repair. Meanwhile, the status of research on MSCs in FIs and the controversies were also discussed. However, the application of MSCs still faces numerous challenges, such as the heterogeneity of cell sources, long-term safety, and feasibility of large-scale production. By analyzing the latest study results, this review intends to offer theoretical support for the application of MSCs in FI treatment and further research.

## 1 Introduction

With multilineage differentiation potential and immunomodulatory properties, mesenchymal stem cells (MSCs) have exhibited great therapeutic potential for various diseases. MSCs, widely present in bone marrow, adipose tissue, oral cavity tissues (Fonticoli et al., 2022) and umbilical cord, are a class of adult stem cells harboring the potential to self-renew and the capacity to differentiate. In addition to multilineage differentiation, strong immunomodulatory properties, i.e., the ability to regulate the activity and function of immune cells, can also be seen in MSCs. Due to these properties, the therapeutic potential of MSCs is great, especially for inflammatory and infectious diseases (Sharma et al., 2021).

Fungal infections (FIs), especially invasive FIs, are a common and serious clinical condition. The incidence of invasive FIs has sharply risen over the last four decades. Recent research revealed that approximately 6.5 million people develop invasive FIs each year, with over 3.8 million deaths, more than 90% of which are attributed to infections by *Aspergillus*, *Candida*, *Cryptococcus*, or *P. carinii*. As a result, invasive FIs have become a major public health problem (Denning, 2024). Fungi are eukaryotic organisms that share many cellular mechanisms with their mammalian hosts, making the development of target drugs difficult. Currently, four classes of antifungal drugs (azoles and polyenes targeting fungal membranes, echinocandins targeting cell walls, and flucytosine targeting RNA/DNA synthesis) are available for invasive mycoses, including novel drugs (voriconazole, posaconazole, and isavuconazole) (Brown et al., 2012). Despite successful use in some FIs, these drugs often fail to be applied promptly to patients due to drug toxicity, high cost, spectrum of activity, and limited bioavailability and route of administration (Denning and Hope, 2010; Puumala et al., 2024). In addition, the development of resistance to some antifungal drugs cannot be neglected, such as multi-drug resistant *Candida* auris (Eix and Nett, 2024).

Due to the restrictions by conventional treatment options and the increase of susceptible populations, fungi have had a worse impact on human health, a major driver of which is a large increase in immunocompromised people with autoimmune diseases, cancers, and post-transplant disorders (Slavin and Chakrabarti, 2012). Considering the increasing resistance to first-line antifungal drugs and the association of fungal diseases with immunocompromised hosts, adjuvant host-directed therapy is recognized as a promising option for a better prognosis, which, as an adjunct to available antifungal therapies, includes cytokine therapy, monoclonal antibody therapy, and cellular immunotherapy. MSCs have received extensive attention due to their low immunogenicity and immunomodulatory plasticity (Williams et al., 2020). Recently, the significant anti-infective effects of MSCs have been verified, which are exerted primarily by releasing antimicrobial peptides (AMPs), disrupting membrane integrity and DNA binding, and suppressing protein synthesis. Therefore, MSCs achieve killing effects against bacteria, fungi, and viruses (Krasnodembskaya et al., 2010; Meisel et al., 2011; Silva-Carvalho et al., 2022; Sung et al., 2016; Yagi et al., 2020). In view of the therapeutic potential of MSCs for invasive mycoses, this study intends to discuss the mechanism of action of MSCs against FIs, the status of clinical research, and the challenges. First, the potential anti-FI mechanism of MSCs was analyzed in detail, including their direct and indirect (by immunomodulation) antifungal effects. Second, the status of clinical research on MSCs was reviewed, and their therapeutic effects across types of FIs were explored. Finally, the challenges faced by MSCs in the FI treatment were discussed. We hope that the findings can offer a theoretical basis and practical guidance for the application of MSCs in the FI treatment and further research.

## 2 Basic characteristics and functional mechanisms of MSCs

### 2.1 Origin and biological properties of MSCs

MSCs are adult stem cells harboring self-renewal and multilineage differentiation potential, which can differentiate into adipocytes, osteocytes, and chondrocytes. The International Society for Cellular Therapy (ISCT) minimal criteria for defining MSCs are as follows: (i) plastic adherence, (ii) specific surface antigen expression (CD73(+)/CD90(+)/CD105(+), CD34(-)/CD45(-)/CD14(-)/other hematopoietic and endothelial markers(-)), and (iii) potential of differentiation into lipoblasts, osteoblasts, and chondrocytes (Dominici et al., 2006).

Widely present in a variety of tissues, MSCs can be isolated from adult and perinatal sources. MSCs of adult origin can be obtained from tissues such as bone marrow, adipose, peripheral blood, dental pulp, synovial membrane and synovial fluid, skeletal muscle, menstrual blood, and skin (Mannino et al., 2022). However, the acquisition of these MSCs is considered invasive and may produce a risk of infection. In contrast, MSCs of perinatal origin (umbilical cord, Wharton jelly, and placenta) are usually harvested noninvasively without ethical issues, demonstrating a greater advantage in clinical studies (Berebichez-Fridman and Montero-Olvera, 2018). Although MSCs from different sources vary in differentiation potential, proliferative capacity, and cytokine profiles secreted, bone marrow-derived MSCs remain the most studied types and have been widely used in animal experiments and clinical trials (Wu et al., 2013). Regardless of the source, MSCs are hypoimmunogenic and lowly or barely express major histocompatibility complex (MHC)-I (e.g., human leukocyte antigen A [HLA-A]) and MHC-II molecules (e.g., HLA-DR), and they also lack the expression of costimulatory molecules such as CD40 and CD80, so they do not activate allogeneic T and B cells, demonstrating a low risk of immunological rejection (Le Blanc et al., 2003; Morandi et al., 2008; Rasmusson et al., 2003). With these properties, MSCs have become an ideal candidate for cellular therapy and regenerative medicine research.

### 2.2 Immunomodulatory functions of MSCs

MSCs have become a promising cellular therapy for various diseases due to their immunomodulatory function, one of their best-known properties. MSCs can restrain the function of T cells, B cells, NK cells, macrophages, and dendritic cells (DCs) by secreting immunosuppressive factors (e.g., transforming growth factor-β [TGF-β], prostaglandin E2 [PGE2], and indoleamine-2,3-dioxygenase [IDO]) and directly interacting with immune cells (Gao et al., 2024; Stevens et al., 2020). For example, MSCs can express PD-L1 to interact with PD-1 on T cells, thus inhibiting T cell activation, and also release immunomodulatory factors by paracrine, thereby relieving inflammatory responses (Gao et al., 2024). Meanwhile, MSCs can regulate the activity of antigen-presenting cells to inhibit the initiation of immune response, achieving immune escape ultimately. In addition, their immunomodulatory effects are highly malleable, i.e., they can alter their behaviors in response to signal changes in the microenvironment (Liu et al., 2022). MSCs prefer a more pronounced immunosuppressive phenotype in the case of acute inflammation, whereas the opposite is the case in chronic inflammation or in the presence of specific antigenic stimuli. It can be seen that MSCs are highly adaptable to the complex and variable environment *in vivo*, and a theoretical basis is provided for the development of more personalized and precise cellular therapies. Research suggests that IDO and nitric oxide (NO) are both key factors for the shift of MSCs between immunosuppression and immunostimulation (Rhee et al., 2015).

### 2.3 Injury repair function of MSCs

MSCs have exhibited great potential in regenerative medicine, which cannot only directly differentiate into specific types of cells but secrete a variety of growth factors, cytokines, and exosomes by paracrine, regulating the local microenvironment and facilitating tissue regeneration (de Morree and Rando, 2023). MSCs exert multilevel therapeutic effects on lung injury. For example, MSCs can suppress the inflammatory response by secreting HGF, IL-10, and TSG-6 in acute respiratory distress syndrome (ARDS) and COVID-19-associated pneumonia, thereby repairing the alveolar structure and reducing pulmonary edema (Fu et al., 2019). Meanwhile, MSCs migrate to damaged tissues through the chemokine signaling axes (e.g., SDF-1-CXCR4) and then differentiate into alveolar epithelial cell type I and type II and lung microvascular endothelial cells, directly replacing damaged cells (Leeman et al., 2019). In addition, MSCs can reduce scar formation by suppressing fibrosis, which ameliorates lung function and facilitates recovery (Han et al., 2023). With a dual role (paracrine regulation and direct differentiation), MSCs have become an ideal candidate for lung injury treatment.

MSCs can be induced *in vitro* to differentiate into neuron-like cells, astrocytes, and oligodendrocytes in central nervous system injury, and can also pass through the blood-brain barrier to achieve a significant repair effect on cerebral infarction and traumatic brain injury. By secreting neurotrophic factors, anti-inflammatory factors, and angiogenic factors, they not only contribute to nerve cell regeneration but also further enhance nerve functional recovery by regulating inflammatory responses and improving the local microenvironment (de Laorden et al., 2023). In addition, the role of MSCs in tissue repair in the cardiovascular system and osteoarticular diseases has been extensively explored. For example, MSCs ameliorate myocardial function post-myocardial infarction by secreting angiogenic and antifibrotic factors (Tan et al., 2020); MSCs differentiate into osteoblasts and regulate the bone microenvironment, thereby facilitating bone healing (Maruyama et al., 2020). These findings indicate that MSCs play important roles in injury repair through diverse mechanisms.

## 3 Features of MSCs that may reduce the severity of FIs

The strong therapeutic potential of MSCs for bacterial infections has been verified, including the ability to directly phagocytose pathogens, secrete antimicrobial peptides (AMPs), and modulate host immune responses and homing to the site of infection, which inspires their application in the treatment of FIs. Research suggests that MSCs can directly phagocytose pathogens (Khan et al., 2017) or secrete AMPs (e.g., LL-37 and β-defensin) to inhibit the growth of pathogens by disrupting their cellular membranes in bacterial infections, which may be equally effective against FIs (e.g., Candidiasis) (Yagi et al., 2020). In addition, MSCs enhance phagocytosis by macrophages and inhibit excessive inflammatory responses by releasing immunomodulatory factors (e.g., IL-10 and TSG-6), thereby protecting host tissues (Galipeau, 2021). Similarly, MSCs with these effects may alleviate the immune imbalance and contribute to the control of inflammation in FIs. By secreting growth factors (e.g., VEGF), MSCs also repair infection-induced tissue damage and accurately home to the site of infection, raising the efficiency of treatment (Ong and Dilley, 2018). Despite few direct studies on MSCs in the field of FIs, their great therapeutic potential for FIs has been evidenced by their application against bacterial infections.

### 3.1 Homing characteristics of MSCs

The efficacy and safety of MSC therapy are affected by several factors, but the “homing” property is the most important. To be specific, MSCs tend to migrate towards the site with high-concentration chemokines, namely the injury site in chronic inflammation, acute inflammation, or no inflammation, and whether they can reach the target tissue depends on the number of them. Saito et al. (2002) first formally introduced the notion of homing to MSCs. Then MSC homing was defined as the arrest of MSCs within the vasculature of the target tissue followed by migration to the target tissue across the endothelium (Karp and Leng Teo, 2009). Numerous research suggests that endogenous or exogenous MSCs are preferentially distributed to the site of injury in the case of ischemia, hypoxia, or injury. Pan WJ’s research team proved the objective existence of stem cell “homing” by *in vivo* imaging of the whole process of stem cell homing (Li D. et al., 2018). Alteration of the microenvironment is the initiating factor of MSC homing, in which chemokines, adhesion factors, and growth factors are locally expressed in tissue injury. Different signaling molecules are secreted in different microenvironments, and some specific ones bind to the corresponding receptors on the membrane of MSCs, thereby driving the homing behavior and directing MSCs to the tissue (De Becker and Riet, 2016). Clinical studies indicated that stem cells can home to injury sites, various organs, and even tumor sites (Table 1; Badri et al., 2011; Chin et al., 2024; Choi et al., 2018; Ikrama et al., 2024; Ju et al., 2017; Liang et al., 2020; Omoto et al., 2014; Schierling et al., 2008; Tashima, 2024; Wang Y. et al., 2022; Weir et al., 2008; Yang et al., 2023; Yeung et al., 2022; Zhuo et al., 2013). To sum up, MSC homing is influenced by a variety of factors, and its time and efficiency may vary due to experimental design and conditions, so more research is required. However, this property provides a solution to the limitations of traditional FIs treatments and adverse drug reactions.

**TABLE 1:** MSC homing in different organs.

Site of homing	Clinical evidence	Clinical challenge
Lung	(1) MSCs can be detected in the lungs post-intravenous injection and disappear on Day 21 (Yeung et al., 2022). (2) Fluorescently labeled MSCs can persist in the lungs for up to six weeks (Weir et al., 2008). (3) MSCs exhibit preferential localization to the region close to alveolar epithelial cell type II (Badri et al., 2011).	The effectiveness of antifungal drugs is limited due to poor tissue permeability, leading to easy relapse (Chin et al., 2024).
Brain	(1) MSCs can pass through the blood-brain barrier (BBB), and remain in the brain for one month post-injection (Tashima, 2024). (2) The homing of MSCs can be observed within 24 h. (3) MSCs can enhance BBB permeability to contribute to drug delivery (Choi et al., 2018).	Antifungal drugs have difficulty penetrating the BBB/BOB/BLB, limiting their efficacy (Ikrama et al., 2024). The risk of toxicity rises due to a narrow therapeutic window (Yang et al., 2023).
Eye	(1) MSCs can migrate to damaged ocular tissues to promote corneal repair (Omoto et al., 2014).	
Ear	(1) MSCs can home to damaged inner ear tissues to promote repair (Ju et al., 2017).	
Liver, spleen	(1) MSCs accumulate mainly in the liver and spleen post-intravenous injection, with a volume of accumulation of 35% in the liver and 8.4% in the spleen on Day 10 (Wang S. et al., 2022).	The efficiency of drug delivery is restricted by the anatomical structure. High toxicity limits the dosage of drugs.
Kidney	(1) MSCs can be detected in the kidney within 7 days post-intravenous or -arterial injection (Zhuo et al., 2013).	
Heart	(1) MSCs can home to the myocardial infarcted area within 1–2 days to promote early repair (Schierling et al., 2008).	
Bone and joint	(1) MSCs can home to the bone graft area to promote repair (Liang et al., 2020).	

Description: Site of homing: The target tissue or organ to which MSCs migrate. BBB, Blood-Brain Barrier; BEB, Blood-Ocular Barrier; BLB, Blood-Labyrinth Barrier. Clinical challenges: Limitations and difficulties of conventional FI treatment in target organs.

### 3.2 Mechanisms by which MSCs may directly inhibit FIs

#### 3.2.1 Phagocytosis

Traditionally, MSCs have been known for their pluripotency, immunomodulation, and tissue repair capabilities, but their phagocytosis has received attention in recent years. Phagocytosis-like functions of MSCs in specific environments, mainly the ability to ingest cellular debris, exogenous particles, or microbes, have been verified, which possess potential biological significance in the regulation of inflammation and immune responses. It has been found that the phagocytosis rate of different particles (latex beads, *E. coli*, *S. aureus*, and *C. albicans*) by human adipose tissue-derived MSCs is 33.8–56.2%, with a mean of 44.37% ± 11.253. Their phagocytic index, despite a high value, is usually lower than that of specialized phagocytes such as macrophages or neutrophils (phagocytosis rate: number of cells phagocytosing fluorescently labeled latex beads/total number of cells; phagocytic index: fluorescence intensity within a single cell) (Costela Ruiz et al., 2022). In bone marrow MSCs activated by a mechanism dependent on TLR2, TLR4, and Dectin-1, fungi may adhere and internalize. This process triggers the expression of inflammatory mediators such as IL-6, IL-17, TNF-α, and TGF-β but has little effect on fungal survival, which may account for the killing effect on pathogens (Rodriguez-Echeverri et al., 2021; Rodríguez-Echeverri et al., 2023). Further investigation is required to determine whether MSCs kill the phagocytized pathogens by fusion of phagosomes with lysosomes like specialized phagocytes, or whether they limit the survival and reproduction of pathogens by internalization.

#### 3.2.2 AMPs

The ability to control the immune response and resist pathogen infections by generating AMPs, one of the biological properties of MSCs, has been a research hotspot recently (Silva-Carvalho et al., 2022). AMPs constitute an important component of innate immunity, with unique advantages (broad-spectrum antimicrobial effect, rapid onset, low drug resistance, immunomodulatory capacity, low toxicity, easily degradable, and adjustable) (Lazzaro et al., 2020). AMPs also exhibit promising efficacy against many multidrug-resistant bacteria (e.g., methicillin-resistant S. aureus) and other refractory infections (e.g., FIs and parasitic infections) (Li et al., 2021). Therefore, they have become important candidates for the treatment of superbugs and stubborn infections in the face of growing drug resistance. AMPs can be directly secreted by MSCs upon encountering pathogens (e.g., bacteria, fungi, and viruses) or when subjected to other immune stimuli (e.g., cytokines and injury signals), which can also be regulated by specific receptors. A variety of immune receptors on the surface of MSCs, such as Toll-like receptors (TLRs) and NOD-like receptors (NLRs), activate the downstream signaling pathways by sensing pathogens and immune signals during infection or inflammation, thereby contributing to the secretion of AMPs (Pezzanite et al., 2021). Moreover, AMPs can be released into the surrounding environment via cellular paracrine or extracellular vesicles (e.g., exosomes), exerting antimicrobial effects (Alcayaga-Miranda et al., 2017).

AMPs are capable of directly destroying the cellular structure of fungi or indirectly controlling FIs by modulating the host immune response (Silva-Carvalho et al., 2022). AMPs can be classified according to their structure, function, and mechanism of action. They mostly can be inserted into the cell membrane of fungi to alter its permeability, leading to leakage of contents and fungal death eventually (Memariani and Memariani, 2023; Vylkova et al., 2007). AMPs can also bind to nucleic acids or proteins in the fungi to inhibit its normal metabolism (Li et al., 2024) or trigger programmed death through the apoptotic pathway (Table 2; Nehls et al., 2020; Shaban et al., 2024; Durnaś et al., 2016; Hsu et al., 2021; Menzel et al., 2017; Wong et al., 2011; Kamysz et al., 2012; Luo et al., 2019; López-García et al., 2006; Kamli et al., 2022; Krishnakumari et al., 2009; Järvå et al., 2018; Costa et al., 2014; Gong et al., 2011; Campbell et al., 2022; Ikonomova et al., 2018; Jang et al., 2008; Moghaddam-Taaheri et al., 2021; Li L. et al., 2018; Hein et al., 2015; Fluckinger et al., 2004; Chen and Lan, 2020; Sebaa et al., 2017; Sowa-Jasiłek et al., 2014, 2016; Yao et al., 2012). In addition to direct antifungal effects, a few AMPs also serve as immunomodulatory molecules to attract immune cells (e.g., neutrophils and monocytes) to the site of infection, or induce the secretion of pro-inflammatory factors (e.g., IL-6 and TNF-α) by the host, thereby synergistically eliminating FIs (Lee et al., 2015). Therefore, the extracted or artificially synthesized AMPs secreted by MSCs can help the development of novel drugs for FIs, especially drug-resistant FIs.

**TABLE 2:** Mechanism of action of AMPs against different species of fungi.

AMPs	Mechanism of action	Target fungi
Cathelicidin/LL-37	Disrupt the integrity of cell wall and membrane, induce oxidative stress, modulate immune responses, and reduce fungal virulence factors	*C. albicans* (Durnaś et al., 2016; Hsu et al., 2021; Menzel et al., 2017; Wong et al., 2011), *A. fumigatus* (Kamysz et al., 2012, Luo et al., 2019), *T. rubrum* (López-García et al., 2006)
β-defensin	Disrupt cell membranes, induce oxidative stress, interfere with cell wall synthesis, inhibit biofilm formation, and enhance host immune responses	*C. albicans* (Kamli et al., 2022; Krishnakumari et al., 2009; Järvå et al., 2018), *C. neoformans* (Costa et al., 2014; Gong et al., 2011), *T. rubrum*
Histatin 5	Bind to cell wall and membrane, induce mitochondrial dysfunction, cause ionic imbalance, mediate oxidative stress by reactive oxygen species (ROS), and inhibit energy metabolism	*C. albicans* (Campbell et al., 2022; Ikonomova et al., 2018; Jang et al., 2008; Moghaddam-Taaheri et al., 2021), other *Candida* species
S100 protein	Chelate metal ions (e.g., zinc and calcium), and limit access to metal ions needed for fungal growth	*C. albicans* (Li L. et al., 2018), *A. fumigatus* (Hein et al., 2015)
Lipocalin-2	Chelate iron chelates (e.g., siderophores) and limit access to iron	*C. albicans*, *Cryptococcus*, *A. fumigatus* (Fluckinger et al., 2004)
Hepcidin	Regulate iron metabolism and limit iron utilization	*C. albicans*, *C. neoformans*, *A. fumigatus* (Chen and Lan, 2020)
Lysozyme	Degrade cell wall peptidoglycan and disrupt the cell wall structure	*C. albicans* (Sebaa et al., 2017; Sowa-Jasiłek et al., 2014, 2016)
PGRPs (peptidoglycan recognition proteins)	Recognize cell wall peptidoglycan, activate immune responses, and inhibit fungal growth	*P. pastoris* (Yao et al., 2012)

Description: 1. Cathelicidin/LL-37 and histatin 5 play prominent roles in directly destroying the structure of fungi, especially *Candida*. 2. S100 protein and lipocalin-2 inhibit fungal growth indirectly by restricting access to metal ions. 3. Lysozyme mainly destroys the fungal cell wall, preventing the spread of fungi. 4. PGRPs and hepcidin, combined with host immunomodulatory functions, exert broad-spectrum inhibitory effects on a variety of fungi. The diverse mechanisms and target fungi of major AMPs are summarized in this table, which can serve as a reference for further research or application.

### 3.3 MSCs modulate the host immune system in ways that could inhibit FIs

MSCs exert an important anti-FI effect by modulating the host immune system, especially for immunocompromised people (Wang Y. et al., 2022). With a potent immunomodulatory capacity, MSCs can promote host immune responses via various mechanisms and effectively suppress FIs. Immunomodulatory plasticity is one of the key properties of MSCs, especially preventing tissue injury due to over-activation of the immune system by suppressing excessive immune responses (Kaundal et al., 2018). In response to different immune environments, MSCs adapt to specific immune responses by adjusting their immunomodulatory functions, such as suppressing excessive immune responses or promoting immune tolerance, thus avoiding damage of the immune system to host tissues or enhancing the body’s immune function. With such plasticity, MSCs are uniquely advantageous for FI treatment (Jiang and Xu, 2020; Liu et al., 2022).

#### 3.3.1 Immunosuppression and immune tolerance

In FIs, the immune system seeks to both eliminate the pathogen and minimize damage to host tissues and organs, suggesting that a tolerance condition is required to avoid harm from excessive inflammatory responses to the host while fighting infections. Such balance is crucial for therapeutic strategies (Weerasinghe et al., 2024), and immunosuppression may be utilized indirectly in some specific cases in the FI treatment. In systemic FIs, an abnormal or over-activated immune response leads to a localized or systemic inflammatory response and even triggers a cytokine storm, especially in immunocompromised or immunodeficient patients. Due to such overreaction, a large number of inflammatory cytokines (e.g., IL-1, IL-6, and TNF-α) will be released, leading to systemic inflammatory response syndrome, and triggering multiorgan failure (Karki and Kanneganti, 2021). MSCs, by secreting anti-inflammatory cytokines (e.g., IL-10, and TGF-β) and directly contacting immune cells, can inhibit the activity of effector T cells, NK cells, B cells, and neutrophils, and reduce the release of pro-inflammatory factors (e.g., TNF-α, IL-1β, and IL-6), attenuating the inflammatory response (Raska et al., 2007).

Some FIs (especially those caused by mold fungi such as *Aspergillus* and *Candida*) may cause an anaphylactic response of the immune system to the fungus and its metabolites. For example, the immune system (e.g., Th2 and Th17) overreacts in allergic bronchopulmonary aspergillosis, and an allergic airway response occurs, causing chronic, recurrent airway inflammation. Persistent inflammation may further worsen infections and immune system burden (Agarwal et al., 2024). MSCs, either by direct contact with T cells or by secreting specific cytokines, can inhibit the activation of Th2 and Th17 cells and reduce immune attacks, while promoting the transformation of pro-inflammatory (M1) macrophages to anti-inflammatory (M2) macrophages. In this way, the immune system becomes tolerant to harmless antigens (e.g., antigens of *Aspergillus*) or autologous tissues, thereby reducing unwanted immune attacks and initiating tissue injury repair, which can effectively alleviate allergic and inflammatory diseases resulting from excessive immune responses (Weerasinghe et al., 2024). Immune responses induced by FIs sometimes excessively damage host tissues, especially in the case of aberrant immunosuppression or immune escape mechanisms. For example, an excessive immune response may lead to inflammatory injury in tissues in cryptococcal meningitis or Candidal sepsis, worsening the condition (Lass-Flörl et al., 2024; Tugume et al., 2023). MSCs can contribute to the development of immune tolerance, helping the body maintain immune homeostasis by avoiding an excessive immune response and damage to vital organs. This process involves regulating the function of DCs and regulatory T cells, making the immune system tolerant to some antigens (Mohammadpour et al., 2015). In addition, as immunosuppression may increase the severity of FIs, caution is needed when using immunosuppressants. MSCs can promote the proliferation and functional recovery of immune cells (e.g., T cells, B cells, and macrophages) by secreting growth factors and cytokines, thereby boosting the immune defense against infections, reducing the infection risk, and ameliorating clinical prognosis.

#### 3.3.2 Promotion of immune cell activity and function

##### 3.3.2.1 MSCs and pattern recognition receptors

A variety of PRRs, such as TLRs and C-type lectin receptors (CLRs), are expressed on the surface of immune cells. TLRs, CLRs, NLRs, and RIG-I-like receptors can recognize different pathogen-associated molecular patterns or damage-associated molecular patterns, and thus activate the immune cell response to infections. With these PRRs, the immune system can effectively recognize the signature molecules (e.g., β-glucan and mannose) in the fungal cell wall to activate the immune response and promote the elimination of the fungus, thereby protecting the host from infections (Brubaker et al., 2015). MSCs can enhance the expression of PRRs on the surface of immune cells by secreting cytokines (e.g., IL-6 and TNF-α). For example, MSCs strengthen the expression of TLR2 and TLR4 by secreting IL-6 (Cortés-Araya et al., 2018), which play key roles in fungal recognition. With enhanced expressions of TLR2 and TLR4, MSCs help immune cells more effectively recognize the components of fungal cell walls (e.g., β-glucan and mannose), thereby initiating an immune response (Rodriguez-Echeverri et al., 2021). Moreover, MSCs enhance the function of PRRs on the surface of immune cells, so that they respond more strongly to fungal invasion, thereby eliminating the fungus by phagocytosis (Kol et al., 2014).

##### 3.3.2.2 MSCs and macrophages

Macrophages are an essential component of the immune system and also a crucial role in FIs. As key cells of innate immunity, macrophages are not only responsible for the early elimination of pathogens but also involved in regulating adaptive immune responses. Through multiple mechanisms, they coordinate the immune response to help the host eliminate fungal pathogens (Casadevall, 2022). After recognizing fungi, macrophages capture and digest the fungal particles by phagocytosis, which, as the primary line of host immune defense, helps prevent the spread of fungi. MSCs facilitate macrophage proliferation, differentiation, and activation through secreting granulocyte-macrophage colony-stimulating factor (GM-CSF). GM-CSF cannot only enhance the phagocytosis of macrophages directly but improve their ability to capture and process fungal particles. In addition, MSCs help maintain macrophage survival and enhance their bactericidal function by secreting M-CSF (Asami et al., 2018). MSCs, by activating the NF-κB and MAPK signaling pathways, further strengthen the phagocytic activity of macrophages (Tomchuck et al., 2008). Cytokines (e.g., IL-6) secreted by MSCs can increase the expression of macrophage surface receptors and thus further promote the phagocytosis and fungal elimination ability of macrophages.

Following infection, macrophages not only directly eliminate fungi by phagocytosis but secrete a series of pro-inflammatory cytokines (e.g., IL-1β, TNF-α, IL-6, and IL-12), biotic factors (e.g., ROS and NO), and enzymes (e.g., lysozyme and acid phosphatase). All these factors contribute to kill or inhibit fungal growth by initiating an acute inflammatory response. In addition, macrophages secrete chemokines (e.g., CCL2 and CXCL8) to attract other immune cells (e.g., neutrophils and T cells) to the site of infection, further enhancing immune defense (Casadevall, 2022). By either secreting or being induced to secrete a variety of inflammatory factors (e.g., IL-6 and TNF-α), MSCs further enhance the immune defense of macrophages, and more ROS, NO, and other biotic factors are produced, thereby enhancing the killing effect of macrophages on fungi (Casadevall, 2022). In addition, MSCs can regulate the phenotype of macrophages and maintain an M1-type phenotype, further enhancing their pro-inflammatory response and ability for the elimination of fungi (Asami et al., 2013). In this way, MSCs cannot only directly participate in fungal elimination but synergize with macrophages to effectively eliminate pathogens by enhancing their immune activity.

##### 3.3.2.3 MSCs and neutrophils

Neutrophils, important effector cells in the immune system, are key roles in the early defense against FIs. They are one of the core cells of the innate immune response, which can rapidly respond to infections and kill and clear the invading pathogens through various mechanisms (Campuzano and Wormley, 2018). MSCs activate neutrophils by secreting IL-6, GM-CSF, and M-CSF, and enhance their proliferation, differentiation, and phagocytosis. In particular, GM-CSF can also stimulate the formation of neutrophil extracellular traps, enabling neutrophils to more effectively capture and digest fungal particles. Moreover, MSCs can enhance the chemotaxis of neutrophils to the site of infection by secreting M-CSF, IL-8, and C5a. With the assistance of these factors, the migration and recruitment of neutrophils are strengthened, so that they can rapidly reach the site of infection and effectively defend against fungi. In addition, MSCs, by secreting pro-inflammatory factors (e.g., TNF-α and IL-1β), can activate NF-κB and MAPK signaling pathways within neutrophils, enhance their oxidative burst, and facilitate the generation of ROS and NO (Rahmani-Kukia et al., 2020), thereby disrupting the cellular structure of fungi and suppressing their growth. In this way, MSCs enhance the direct fungal clearance ability of neutrophils.

##### 3.3.2.4 MSCs and DCs

DCs are important antigen-presenting cells capable of transmitting information of exogenous pathogens to T cells and initiating specific immune responses. MSCs contribute to the maturation and activation of DCs by secreting cytokines (e.g., IL-6, TNF-α, and IL-1β). In cases of FIs, mature DCs can more efficiently ingest and process pathogens and activate T cells by presenting antigens via MHC molecules. MSCs can increase the expression of MHC II on the surface of DCs, enhancing their antigen-presenting capacity (Ryu et al., 2015). In this way, DCs can more effectively recognize and present fungal antigens to CD4^+^ T cells, thereby eliciting strong immune responses.

##### 3.3.2.5 MSCs and NK cells

NK cells are key cells for antiviral and antitumor activity and also play an important role against FIs. MSCs stimulate the activation of NK cells by secreting cytokines (e.g., IL-2, IL-15, IL-12, and TNF-α). These factors can enhance the cytotoxicity and fungal-killing effect of NK cells. For example, IL-2 and IL-15 can strengthen the proliferation and activation of NK cells and enhance their immune response in FIs. Interferon-γ (IFN-γ) and IL-12, by activating receptors on the surface of NK cells (e.g., NKG2D and NKp46), enhance the recognition ability of NK cells for FIs, and stimulate their secretion of effector molecules (e.g., perforin and granzyme), directly killing fungi or infected cells. In addition to cytotoxicity, NK cells also eliminate pathogens through phagocytosis. MSCs enhance the phagocytosis of NK cells by secreting GM-CSF and IL-12, enabling them to more efficiently capture and eliminate fungal pathogens (Asami et al., 2013).

##### 3.3.2.6 MSCs and adaptive immunity

Adaptive immunity in FIs is a complex and multilevel process. A more elaborate immune response is usually required against FIs than viral or bacterial infections because fungi are usually multicellular organisms with complex structures and life cycles. Adaptive immunity, involving B cells, T cells, and the resulting antibodies, are important factors against FIs. Although innate immunity is more critical in the early defense against infection, adaptive immunity is critical in eliminating the infection and preventing recurrence.

The Th1 response is particularly important for the clearance of fungi such as *Candida*. Th1 cells enhance the bactericidal function of macrophages by secreting IFN-γ, helping clear intracellular fungi. Th17 cells promote the recruitment of inflammatory cells and local immune responses by secreting IL-17, thereby clearing exogenous fungi (e.g., *C. albicans*) (Niu et al., 2018). In adaptive immunity, B cells produce antibodies by recognizing fungal surface antigens, which help either neutralize or label the fungus for recognition by other immune cells. The antibodies exert strong effects against some types of fungi (for example *Candida*).

By binding to fungal antigens, antibodies produced by B cells can form immune complexes that can be recognized and cleared by macrophages and neutrophils (Mukaremera and Nielsen, 2017). MSCs work in adaptive immunity by the immunomodulation of T and B cells, and they can secrete IL-6 and IFN-γ in response to FIs to stimulate differentiation of Th1 and Th17 cells (Shi et al., 2023), two cells crucial for the clearance of FIs. For example, Th17 cells recruit immune cells by secreting IL-17, enhancing local immune responses, which are particularly critical against FIs. MSCs enhance the function of antigen-presenting cells (e.g., DCs) and promote the formation of memory T cells (Luque-Campos et al., 2019). The resulting memory immune response is important for defense against FIs in the future. Finally, antibodies not only help neutralize the fungus but label the fungus for recognition by other immune cells. For example, MSCs may promote B cell differentiation by secreting cytokines (e.g., IL-4 and IL-21), thereby promoting the production of antifungal antibodies (Dabrowska et al., 2020).

## 4 Experimental evidence of the effects of MSCs on FIs

In recent years, MSCs have received widespread attention for their potential use in cellular therapy for fungal infectious diseases, as shown in Table 3.

**TABLE 3:** Effects of mesenchymal stem cells (MSCs) in various fungal diseases and experimental models.

Cell type	Fungal species	Disease type	Experimental model	Key findings	Source
IL-17^+^ and IL-17^–^ MSC subpopulations	*C. albicans*	Candidiasis	*In vitro* co-culture model;C57BL/6 mouse infection model	1. IL-17^+^ MSCs significantly inhibit the growth of *Candida albican*s, likely via IL-17 secretion.2. IL-17^+^ MSCs exhibit impaired immunoregulation compared to IL-17^+^ MSCs.3. IL-17 downregulates TGF-β1 via NFκB, impairing immunomodulatory functions.4. IL-17^+^ MSCs show superior fungal clearance.	(Yang et al., 2013)
Human MSCs	*A. fumigatus*	Invasive aspergillosis (IA)	*In vitro* co-culture model; immune cell functional assay	1. MSCs phagocytose *A. fumigatus* spores but show no direct effects on hyphae.2. Co-culture increases IL-6 expression but not TNF-α or IFN-γ.3. MSCs do not impair T cell activation or function.4. MSCs do not alter oxidative burst activity of phagocytes.	(Schmidt et al., 2017)
Mouse MSCs (mMSCs)	*A. fumigatus*	IA	*In vitro* co-culture model; immune cell functional assay	1. mMSCs reduce TNF-α secretion while increasing IL-10 in J774A.1 macrophages.2. mMSCs inhibit spore growth; GM-CSF weakens this effect.3. mMSCs suppress NFκB translocation, indicating anti-inflammatory effects via Toll-like receptor pathways.	(Cho et al., 2016)
Mouse bone marrow MSCs	*A. fumigatus*	Th17-mediated allergic airway inflammation (AAI)	Acute and recurrent AAI models; intravenous MSC injection in mice	1. MSCs mitigate airway hyperresponsiveness (AHR) and reduce airway resistance and neutrophil counts in acute AAI.2. MSCs reduce IL-17a and IL-6 in airway fluid during acute AAI.3. MSCs show limited effects in recurrent AAI.4. Effects may involve suppression of Th17 pathways.	(Lathrop et al., 2014)
Bone marrow MSCs(BM-MSCs)	*P. brasiliensis*	Chronic pulmonary mycosis (PCM)	Intranasal inoculation of BALB/c mice; intravenous BM-MSC injection	1. BM-MSCs exacerbate granulomatous inflammation and fibrosis.2. Increased fibroblast count and soluble collagen levels.3. Upregulation of fibrosis-related genes (e.g., Col3α1, TGF-β3).4. Combined itraconazole therapy reduces fibrosis markers.	(Arango et al., 2017)
BM-MSCs	*P. brasiliensis*	Chronic pulmonary mycosis (PCM)	Intranasal inoculation of BALB/c mice; intravenous BM-MSC injection	1. BM-MSCs aggravate inflammation with increased neutrophils, eosinophils, and M2 macrophages.2. Elevated fungal burden in lungs and spleen.3. Increased pro-inflammatory cytokines (e.g., IL-6, GM-CSF).4. Reduced Tregs and Th17 cells.	(Arango et al., 2018)
BM-MSCs	*P. brasiliensis*	Chronic pulmonary mycosis (PCM)	*In vitro* co-culture model; TLR and Dectin-1 blocking experiments	1. BM-MSCs recognize *P. brasiliensis* via TLR2, TLR4, and Dectin-1.2. Activated BM-MSCs secrete IL-6, IL-17, TNF-α, and TGF-β.3. TLR blocking reduces cytokine expression.4. BM-MSCs exhibit pro-inflammatory properties and lack antifungal efficacy.	(Rodriguez-Echeverri et al., 2021)
BM-MSCs	*H. capsulatum*	Histoplasmosis	*In vitro* co-culture model; TLR and Dectin-1 blocking experiments	1. *H. capsulatum* upregulates TLR2 expression in MSCs.2. MSCs phagocytose *H. capsulatum* but lack fungicidal activity.3. Infection induces IL-6 but suppresses other cytokines (e.g., IL-17, TNF-α).4. *H. capsulatum* inhibits MSC proliferation and induces apoptosis.	(Rodríguez-Echeverri et al., 2023)
Umbilical MSCs (uMSCs)	*F. oxysporum*	Fungal keratitis (FK)	Corneal scratch model; natamycin treatment followed by subconjunctival uMSC injection	1. uMSCs reduce corneal scar area and opacity scores.2. Restored collagen fiber structure.3. Downregulated fibrotic factors (e.g., α-SMA, TGF-β1).4. Improved corneal transparency and minimized scarring.	(Zhou et al., 2019)

Yang et al. (2013) demonstrated for the first time the efficacy of MSCs against *C. albicans* infection using IL-17^+^ MSCs, including direct growth inhibition of *C. albicans* in *in vitro* experiments and therapeutic effect on *C. albicans*-infected mice. During this process, IL-17 played a major role and suppressed *C. albicans* in a dose-dependent way, and MSCs were stronger than CM in inhibiting the growth of *C. albicans*. This suggests that the inhibitory effect of MSCs on *C. albicans* partly originates from the soluble molecules secreted. Given the great potential of AMPs in antifungal therapy, a direction for future research is presented.

Consistent results have been obtained in the research on the regulatory effect of MSCs on immune responses against FIs. Schmidt et al. (2017) found that MSCs can influence the body’s immune response to *A. fumigatus* ellipsoidal variant by modulating cytokine responses, exhibiting their potential in antifungal immunity. Cho et al. (2016) also noted that MSCs can suppress the inflammatory response to a certain extent and attenuate the cytokine release induced by FI, indicating that MSCs may enhance the body’s resistance to fungi by inhibiting the production of pro-inflammatory cytokines. Consistent with the findings of Lathrop et al. (2014) et al. pointed out that MSCs can significantly inhibit Th17-mediated inflammatory responses in an allergic airway inflammation model, further verifying the role of MSCs in regulating immune responses. Meanwhile, Zhou et al. (2019) demonstrated that umbilical cord-derived MSCs can help attenuate corneal scarring post-FIs, further highlighting the potential of MSCs in antifungal therapy. To sum up, MSCs may improve the outcome by modulating the host immune environment in response to FIs, particularly by regulating the pro-/anti-inflammatory cytokine balance.

Although the therapeutic potential of MSCs for FI has been verified in many studies, significant differences are present in these study results. For example, Arango et al. found in an FI-induced pulmonary fibrosis model that MSCs transplanted worsen chronic inflammatory responses and lung fibrosis rather than displaying an anti-fibrotic effect. They observed that in mice treated with MSCs, the area of granulomatous inflammation and the degree of fibrosis in the lungs significantly increased, and the number of fibroblasts and dissolved collagen rose in the lungs. The results are attributed to the possible synergy of MSCs with the infection-induced inflammatory response as well as the direct interaction of MSCs with fungi that may contribute to collagen overexpression and tissue remodeling, contrary to the anti-inflammatory effects of MSCs demonstrated in other studies (Arango et al., 2017). Such a discrepancy may be attributed to different experimental models and types of infection. In addition, MSCs transplanted in a model of *P. brasiliensis* infection lead to an increase in fungal load and exacerbation of lung inflammation (Arango et al., 2018), suggesting a complex role of MSCs in certain cases. Rodriguez-Echeverri et al. (2021) further noted that in the case of *P. brasiliensis* infection, MSCs are activated and produce a series of pro-inflammatory factors, worsening the inflammatory response. The above findings are contrary to the idea in previous studies that MSCs inhibit inflammation, suggesting that MSCs may have no single effect in some specific contexts and may be influenced by the pathogen. In addition, Rodríguez-Echeverri et al. (2023) argued that *H. capsulatum* infections also affect the proliferation and differentiation of MSCs, further impairing their immunomodulatory capacity. This also suggests different effects on the function of MSCs across FIs. To sum up, these differences may be closely related to the experimental design, infection model, source of MSCs, and characteristics of pathogens, so in-depth studies are needed in the future.

## 5 The future of MSCs as a therapy for FIs

In the future, research should focus on exploring more therapeutic potential of MSCs for FIs, especially on new sources of stem cells and combination therapies. According to available studies, MSCs exert some immunomodulatory and anti-inflammatory effects against FIs, but many issues remain unresolved and we need to gain a deeper understanding of the mechanisms of MSCs in FIs. Meanwhile, many challenges and obstacles are faced when MSCs are applied to the clinical treatment of FIs. First, the sources of MSC and the standardization of the preparation process remain an urgent issue. MSCs from different sources (e.g., bone marrow and umbilical cord) may differ in function and properties, so unified standards of preparation and application must be developed before clinical application. In addition, whether the existing preparation techniques and cell expansion methods are mature may affect the function of MSCs, making it difficult to guarantee stable efficacy in practice. Second, the immunomodulatory properties of MSCs, although beneficial in some cases, may trigger adverse reactions in the case of FIs. For instance, MSCs may suppress the immune response of macrophages, thereby affecting the body’s resistance to fungi, in which case the risk of exacerbation or recurrence is produced, seriously affecting the patient health. Therefore, it is necessary to fully evaluate the potential risks and benefits of MSCs before application. Finally, the design and ethical review of clinical trials is also a key issue. Due to the innovative and uncertain nature of MSC therapies, related ethical and legal issues will be an obstacle to clinical popularization. Researchers need to work closely with Ethics Committees to ensure that the study protocol meets ethical requirements; patients should be given full informed consent to protect their rights and interests.

In summary, MSCs have exhibited some therapeutic potential for FI, but multiple challenges remain to be overcome during clinical translation.
